# Realizing the right to health in Latin America, equitably

**DOI:** 10.1186/s12939-020-01332-y

**Published:** 2021-01-13

**Authors:** Manuela Villar Uribe, Maria-Luisa Escobar, Ana Lorena Ruano, Roberto F. Iunes

**Affiliations:** 1 World Bank Group, Washington DC, USA; 2 Independent Consultant, Washington DC, USA; 3 Center for International Health, Department of Global Public Health at University of Bergen, Bergen, Norway; 4 Center for the Study of Equity and Governance in Health Systems (CEGSS), Guatemala City, Guatemala

**Keywords:** Right to health, Universal health coverage, Latin America and Caribbean, Equity

## Abstract

This special issue “Realizing the Right to Health in Latin America and the Caribbean” provides an overview of one of the most challenging objectives of health systems: equity and the realization of the right to health. In particular, it concentrates on the issues associated with such a challenge in countries suffering of deep inequity. The experience in Latin America and the Caribbean demonstrates that the efforts of health systems to achieve Universal Health Coverage are necessary but not sufficient to achieve an equitable realization of the right to health for all. The inequitable realization of all other human rights also determines the realization of the right to health.

## Introduction

Latin America’s renewed commitment to Universal Health Coverage [9, 22], the explicit constitutional recognition of the right to health in much of the region [8], the persistent efforts to increase financial protection for health events [3] in a context of a pervasive income inequity, makes Latin America’s experience in the pursuit of the progressive realization of the right to health, quite relevant globally. Although the region has seen improvements in ensuring the right to health [6] and increasing Universal Health Coverage for its population [13, 19], persistent inequalities have highlighted barriers to achieving these dual goals.

Latin America and the Caribbean encompasses a large variety of experiences in its process of the realization of the right to health for all. However, the region’s achievements and challenges either at the sub-national, national or regional levels [11] are not always documented. This special issue acknowledges the continuing path towards the realization of the right to health and thus, seeks to present and promote a deeper and more nuanced understanding of this path by presenting a variety of examples related to equity in health from countries across the region. The discussion here presented gains even further relevance in the context of the challenges to the rights of Latina American citizens brought by the COVID-19 pandemic.

A summary of 13 papers included in this special issue is presented here to highlight the results of the ongoing challenges in the Latin America and the Caribbean Region to ensure the equitable realization of the right to health enshrined in their constitutions. There are four types of studies: First, those presenting inequalities in the performance outcomes of health systems in relation to efforts to ensure the right to health for vulnerable populations [4, 5, 17]. Second, studies highlighting efforts to ensure the right to health for specific disease conditions [10, 12, 20, 24]. Third, papers analyzing the role of judicialization as a mechanism to ensure the right to health of populations and what it is currently playing across some of the countries in the region in this area [2, 16, 23, 26]. Fourth, the summary presents the studies that try to elicit underlying policy factors to the equitable realization of the right to health across countries [14, 15].

### Selection process for articles included in special issue

With the aim of ensuring a multiple-perspective approach to the process towards an equitable realization of the right to health, the articles included in this special issue were selected among submissions of research abstracts presented by researchers from countries across Latin America and the Caribbean. The call for abstracts drew on the broad network of researchers and policy makers of the *SaluDerecho*^1^ initiative, a regional effort supported by the World Bank, that promotes dialogue and learning on the right to health. Abstracts were selected to represent various country contexts and perspectives on the right to health as follows: Right to health and specific health care process; right to health and population groups; right to health and health conditions; right to health and current mechanisms used to ensure its realization; and, proposed interventions to reduce or mitigate inequalities in the realization of the right to health for all.

### Findings

Articles in this series begin by presenting inequalities in the basic principles of universal health coverage and the right to health. The authors, [5] explore available data from Haiti to assess inequalities in health service utilization, catastrophic health expenditures and the determinants of health seeking behavior in the years 2012 and 2013 after the devastating 2010 earthquake. The authors find increases in catastrophic health expenditures between the 2 years, mostly driven by increases in these expenditures among the poor and at a time of a decrease in external donor funding, when compared to those observed in the period immediately after the earthquake. The findings also show greater catastrophic household expenditures among the poor as compared with those of the wealthier households, although the results also suggest persons who sought care from community health workers were less likely to incur in catastrophic health expenditures as compared to those receiving care from other types of providers [5]. These results suggest that models of care could be playing a role in out-of-pocket health care expenditures in Haiti.

Two articles explore inequalities in access and continuum of care, highlighting the cases of breast and cervical cancer in Brazil. Despite the constitutional mandate of the right to health for all in the country, [17] found important differences among women of different income groups. Using mixed methods, the authors gained insights into the variation in the organization of breast and cervical cancer care across Brazil. Drawing from an analysis of nationally representative data, a review of key documents, observations and interviews with health workers, the authors find lower rates of screening in poorer states and among women of the lowest income brackets, low rates of diagnosis of breast and cervical cancers (26 and 29% respectively) and high waiting times for initiation of treatment for patients diagnosed outside the hospital setting. Moreover, those who follow the recommended treatment pathways, restricted to the public sector, also experience high waiting times to initiate treatment despite the legally mandated maximum waiting time of 60 days [17].

Compare narratives of women of different levels of education and socio-economic status who have sought care with private and public providers for breast cancer in the city of Belo Horizonte in Brazil [4]. Using in-depth interviews, the researchers elicit differences in the pathways to initiation and continuation of treatment for their illness. This study finds differences in breast cancer care between participants that may be associated with prevailing social inequalities such as schooling, income, and ethnicity, resulting in lower quality of care for women of greater vulnerability. The findings include greater preventive care behaviors among more educated women, lower waiting times for consultations among more educated women with private health insurance. It also finds greater barriers to access care, including delays that drew women of lower education levels to seek care outside of the public system and incur high out-of-pocket expenditures. Women with lower levels of education were less able to rely on informal networks that expedite diagnostic and treatment procedures. Finally, highlighting the complexities in the demand and supply side barriers to the realization of the right to health, the findings suggest stark differences in the exercise of the right to information and involvement in the definition of the treatment protocol for their individual case. The study finds that wealthier women exercised their right to choose their treatment type and less wealthy women were given few opportunities to dialogue on their treatment options [4].

Although evidence from Brazil points to stark inequalities for women of different socio-economic status, in Ecuador, one article points to nuanced effect that the evolution towards the realization of the right to health could have on equity [12]. Analyzed inequalities in sexual and reproductive health indicators in 2009 and 2015 across different provinces. Their findings suggest differences in changes in equity for the different indicators investigated. The authors found improvements in equity for indicators of adolescent fertility, and report on growing inequalities in home deliveries and maternal mortality between wealthy and poorer provinces. The authors found no significant changes in equity for indicators of obstetric and abortion complications and C-sections among provinces with greater percentages of population in poverty than wealthier provinces [12].

Inequity in the realization of the right to health is particularly relevant among vulnerable populations [10]. Reviewed information from policy and legal documents as well as in-depth interviews with health workers and authorities to examine the barriers faced by indigenous people and rural settlers in the Peruvian Amazon for obtaining tuberculosis (TB) diagnosis and appropriate treatment. The authors find a strong legal framework in place to protect the right to health of indigenous people and people affected by TB. However, structural factors such as misaligned incentives and motivation for health workers, overall scarcity of human resources in rural health facilities, lack of support for training and outreach activities, lack of necessary supplies for diagnosis and transportation of samples and the misdirected use of available inputs to low risk populations, contribute to delays in detection, diagnosis and TB treatment [10]. This is an example where having legal developments around the right to health but not enough or adequate implementation of supply-side interventions to improve the delivery or care is of course not conducive to an equitable realization of the right to health for all [20]. Used qualitative methods to explore the experiences of rehabilitation care of adults with physical disabilities in Colombia who came from different socio-economic backgrounds. This research highlights large barriers for the care of disabled adults in the country. The authors find that these patients face challenges to access health services related to their personal mobility, perceptions and knowledge of providers on disability management and lack of support to navigate the health system for obtaining care for their specific needs [20].

One article points to the life-course effects of socioeconomic vulnerabilities. In an analysis of a cross-sectional survey of Mexican immigrant women in New York City, [24] explore the correlation between short relative leg length, resulting from childhood undernutrition and infections, with their current state of overweight or obesity. The findings of this study point to an important relationship between the effects of early life socio-economic conditions and the adverse risk of overweight and obesity later in life [24]. This points to the central role that the realization of the right to food, during all life stages, and the right to decent housing and living conditions (outside the direct realm of health systems) play in preserving inequity in health later in life.

Judicialization in health care is a phenomenon where the Judicial branch intervenes in order to enforce the realization of the right to health. It has appeared as a response of patient’s legal action (less often of whole communities) to obtain care. “Lawsuits” to obtain care that has been denied, have been used by patients across countries in the Latin America and Caribbean Region to different extents. In a scoping review [2], provide a background on the existing evidence on the impact of judicialization on equity as published in peer-reviewed journal articles, as well as grey literature. Their findings suggest a concentration of studies about judicialization cases in Brazil (54% of studies) and Colombia (23% of studies). They also find an increase in publications on the subject over time with a greater percentage of publications between 2011 and 2014 (66%) as compared to the previous 4-year period (17%) and the following 4 years after 2014 (17%). Although a third of studies have ambiguous findings on the effect of judicialization on equity (31%), nearly half (49%) point to negative effects on equity (increases in inequity), and 20% suggest improvements in equity due to this mechanism [2]. In support of these findings [26], outline variation in the use of judicialization across Latin America and the Caribbean to enforce the right to health while balancing the democratization and legitimization of priority-setting processes. Authors argue the importance of transparency in the priority setting process and communication about the explicit guarantees of each system for an adequate regulation to be ensured [26].

Contributing to the limited available knowledge on the use of judicialization [16], explore the relationship between the use of judicialization of health care in Minas Gerais, Brazil, and the Human Development and Social Vulnerability indexes of municipalities of beneficiaries who use this grievance mechanism to demand their right to health. The authors find that residents of municipalities with Higher Development Indexes and lower Vulnerability indexes are more likely to file lawsuits in demand for their rights to health services [16].

Using qualitative methods [23], compare the use of judicialization for access to medicines in Argentina, Brazil, Colombia, and Chile. The study findings show that, in the four countries, the extensive use of the judicialization mechanism to guarantee the constitutional right to health arose from the difficulties in guaranteeing access to medicines included in insurance schemes as well as the influence of the pharmaceutical marketing on priority setting processes and prescription behaviors. The study finds that judicialization has served as a mechanism of accountability from facility and insurance managers to patients, however the mechanism has also brought about the financing of medicines and technologies without evidence of their efficacy or safety. Finally the authors report finding that judicialization has had an impact on research and development policies while in Colombia it has encouraged the recognition of the right to health as a fundamental right and the development of price control policies for certain medicines [23].

As referred by authors of papers in this issue, less educated citizens, more vulnerable populations, those with less access to information, and people in countries with inefficient and cumbersome legal systems have less access to the Judicial system to demand their right to health. Research on the relationship between equity and judicialization is limited and remains inconclusive. Further research is necessary to evaluate the impact of judicialization on equity in the realization of the right to health.

Judicialization has begun to be complemented by upstream mechanisms that could aim to reduce, upstream, potential inequalities within and across countries in the Latin America and Caribbean Region [14, 15]. Used a participatory research approach with representatives from eight countries in Latin America and South Korea to explore the role of confidentiality agreements as a tool for market regulation and recommendations for their use. The approach included representatives from all three branches of government having health policy makers, members of the judicial and the legislative, as well as other health system representatives like practitioners, insurers, patient organizations and academics from the different countries. The study identified barriers to the equitable negotiation of drug prices across countries. Lack of transparency in pricing and the resulting asymmetries in information are safeguarded and amplified by existing confidentiality agreements. This paper calls for international regulation, so all countries have access to the same information and not restricted by confidentiality agreements. The stakeholders participating in the research suggest the establishment of standardized processes agreed across countries, for the negotiation of drug prices that replace confidentiality agreements and foster transparency of drug prices and improve cross-country equity [14, 15].

Additionally, the pros and cons of continuity of payment for post-trial care, was explored by [14, 15], also through a participatory research approach. Mechanisms ensuring the continuity of post-trial health care treatments, preventing financial ruin of individuals and families participating on clinical trials, have been proposed in the region for progress towards an equitable realization of the right to health. The final consensual recommendation was a World Health Organization resolution recommending all countries call for the medicine and medical device industries, governments, or the sponsors of the clinical trials, to ensure the continuity of care and prevent cross-country inequity as well as detrimental effects on patients and their families once trials end [14, 15].

## Discussion

The papers included in this special issue provide a perspective of the process of the realization of the right to health across a variety of country contexts. It includes cases in countries with extensive health insurance coverage such as Colombia and Brazil and where healthcare litigation is common. It provides some contrast in country contexts of varying levels of wealth and of inequalities while also presenting cases related to non-communicable illnesses (cancers), reproductive health care, infectious diseases and nutrition.

Although the perspectives vary, the research papers in this issue portray a pathway towards the realization of the right to health that is not always linear nor strictly equitable. The realization of the right to health for all citizens is a process that is imbedded in the structural inequity of the societies in question and as such, impacted by it. Such inequity expresses itself in the inequitable realization of all other human rights and consequently also determine the realization of the right to health. The research papers exploring processes and outcomes of the realization of the right to health, show a common theme of inequalities in the quality of care received by the wealthy compared to the poor and for those in conditions of vulnerability due to ethnicity or residence. We see that the realization of the right to health is complex, sometimes apparent in the processes of care, such as waiting times and continuity, sometimes differing across indicators of health services and related outcomes and sometimes persistently inequitable across a person’s life-course.

Health policies directed to improve availability, access, affordability and quality of health care have rendered positive results in the region. The Latin America and the Caribbean region has introduced numerous policies and programs to improve not only public health, but both the supply and demand of health care services. Efforts to improve the supply of health care services have sought to expand the availability and quality of services. Creating and equipping more facilities across the national territory and improving the hours of service, increasing the numbers of human resources for health, developing stronger supply chains for better availability of medical inputs and pharmaceuticals, as well as developing policies to control their prices and improve their procurement, are examples of such efforts. In addition, many countries in Latin America and the Caribbean have also introduced reforms to their health systems in search of an expansion in health coverage by introducing demand-side interventions to improve access to care, over the last 30 years. Among those there are conditional cash transfer programs, subsidized health insurance, vouchers and other mechanisms to improve financial protection [18].

Those efforts to target supply and demand barriers to the realization of the right to health have been combined with the recognition of the right to health as a human right in national Constitutions and the introduction of legal mechanisms to enforce its fulfillment. The combined action in all fronts has generated two type of results: on one hand, access to health care services has improved and on the other hand inefficiencies in the delivery of care are more evident, making inequity impossible to dismiss. Constitutions in most countries in Latin America and the Caribbean, provide a legal framework that emphasizes the country’s commitment to respect, protect and fulfill human rights and explicitly the right to health. Overtime, jurisprudence around the right to health has evolved making clearer and more concrete the government’s responsibilities to fulfill the right to health [21]. In many cases, countries have provided legal instruments intended to enforce the realization of the right to health, while empowering and giving voice to citizens to demand government’s accountability. As we see in the papers in this issue, moving towards health coverage in Latin America and the Caribbean region provides multiple examples evidencing inequity in the realization of the right to health and even in the presence of the constitutional mandate to the right to health.

The phenomena of enforcing the realization of the right to health via judicial intervention has made the already existing inequalities more evident and highlight health system inefficiency. There is limited knowledge on the impact of judicialization on equity. As referred by authors of papers in this issue, there is unequal access to the judicial system and research on the relationship between equity and judicialization is limited and remains inconclusive, however, unequal access to the judicial system is not a direct cause of inequity in the realization of the right to heath.

More data and new research are necessary to disentangle the relationship between equity, judicialization and the realization of the right to health. Robust quantitative evaluation methods would prove useful to evaluate causality and to shine more light on the impact of judicialization on equity. On the other hand, variation in the enforcement of guarantees and the use of judicialization mechanisms is seen to be intertwined with the legitimization and transparency of the priority-setting processes.

Overall, these papers highlight the importance of understanding that the realization of the right to health for all citizens is a process imbedded in the structural inequity of the societies in question. Structural inequity determines progress on and challenges the path to the realization of the right to health, even with enforcement of a constitutional mandate through judicialization. Despite all these efforts, the search for universal coverage continues to this day with the underlying promise to fulfill the right to health for all citizens and continues to be most challenged in societies with pervasive income inequity.

## Conclusion

The experience in Latin America and the Caribbean demonstrates that health systems’ efforts are necessary but not sufficient to ensure an equitable realization to the right to health. This is a process intertwined, dependent on, and one that progresses in tandem with the realization of a whole set of other human rights. This is of particular importance in societies plagued with deep inequity [7, 25]. The right to obtain an education, live in decent housing, the right to food and to land, the right to a safe environment, to information, as well as many others are not independent of each other, and certainly define the results of the realization of the right to health [1]. That is why in order to achieve an equitable realization of the right to health for all, a multi-systemic approach is necessary. Multiple efforts on the part of health systems, and even in the presence of mechanisms to enforce the constitutional mandate to the right to health, are necessary but not sufficient to achieve an equitable realization of the right to health.

## Data Availability

Not applicable.

## Footnotes

1 See: https://saluderecho.net/
